# p38 MAPK Facilitates Crosstalk Between Endoplasmic Reticulum Stress and IL-6 Release in the Intervertebral Disc

**DOI:** 10.3389/fimmu.2018.01706

**Published:** 2018-08-17

**Authors:** Olga Krupkova, Aleksandra Sadowska, Takuya Kameda, Wolfgang Hitzl, Oliver Nic Hausmann, Juergen Klasen, Karin Wuertz-Kozak

**Affiliations:** ^1^Institute for Biomechanics, ETH Zurich, Zurich, Switzerland; ^2^Fukushima Medical University, Fukushima, Japan; ^3^Biostatistics, Research Office, Paracelsus Medical University, Salzburg, Austria; ^4^Department of Ophthalmology and Optometry, Paracelsus Medical University, Salzburg, Austria; ^5^Neuro and Spine Center, Hirslanden Klinik St. Anna, Lucerne, Switzerland; ^6^Prodorso, Zurich, Switzerland; ^7^Academic Teaching Hospital, Spine Research Institute, Paracelsus Medical University, Salzburg, Austria; ^8^Spine Center, Schön Klinic Munich Harlaching, Munich, Germany; ^9^Department of Health Sciences, University of Potsdam, Potsdam, Germany

**Keywords:** intervertebral disc, inflammation, endoplasmic reticulum stress, p38 MAPK, CHOP, GADD153, GRP78, IL-6

## Abstract

Degenerative disc disease is associated with increased expression of pro-inflammatory cytokines in the intervertebral disc (IVD). However, it is not completely clear how inflammation arises in the IVD and which cellular compartments are involved in this process. Recently, the endoplasmic reticulum (ER) has emerged as a possible modulator of inflammation in age-related disorders. In addition, ER stress has been associated with the microenvironment of degenerated IVDs. Therefore, the aim of this study was to analyze the effects of ER stress on inflammatory responses in degenerated human IVDs and associated molecular mechanisms. Gene expression of ER stress marker GRP78 and pro-inflammatory cytokines IL-6, IL-8, IL-1β, and TNF-α was analyzed in human surgical IVD samples (*n* = 51, Pfirrmann grade 2–5). The expression of GRP78 positively correlated with the degeneration grade in lumbar IVDs and IL-6, but not with IL-1β and TNF-α. Another set of human surgical IVD samples (*n* = 25) was used to prepare primary cell cultures. ER stress inducer thapsigargin (Tg, 100 and 500 nM) activated gene and protein expression of IL-6 and induced phosphorylation of p38 MAPK. Both inhibition of p38 MAPK by SB203580 (10 µM) and knockdown of ER stress effector CCAAT-enhancer-binding protein homologous protein (CHOP) reduced gene and protein expression of IL-6 in Tg-treated cells. Furthermore, the effects of an inflammatory microenvironment on ER stress were tested. TNF-α (5 and 10 ng/mL) did not activate ER stress, while IL-1β (5 and 10 ng/mL) activated gene and protein expression of GRP78, but did not influence [Ca^2+^]_i_ flux and expression of CHOP, indicating that pro-inflammatory cytokines alone may not induce ER stress *in vivo*. This study showed that IL-6 release in the IVD can be initiated following ER stress and that ER stress mediates IL-6 release through p38 MAPK and CHOP. Therapeutic targeting of ER stress response may reduce the consequences of the harsh microenvironment in degenerated IVD.

## Introduction

Low back pain (LBP) is the leading cause of disability, activity limitation, and lost productivity throughout the world today, with approximately 80% of all people suffering from back pain at least once in their life (1). Degenerative disc disease (DDD), a progressive multifactorial disorder of intervertebral disc (IVD), often caused by inflammation and subsequent irritation of spinal nerves, is one of the main factors in the development of LBP (1). Despite the significant socio-economic consequences associated with DDD, no therapy can presently stop or reverse the degenerative process (2, 3). Current treatments are still only symptomatic, mainly comprising of general pain medication and spinal surgeries (4). Success of novel biological therapies (e.g., stem cell implantation) is often hindered by the pre-existing harsh inflammatory microenvironment in the degenerated IVD (5, 6). Nevertheless, mechanisms underlying the degenerative microenvironment in the IVD are not yet sufficiently understood.

DDD is characterized by a catabolic shift in IVD cells and a decline in extracellular matrix, altering load distribution and predisposing to LBP. LBP can raise both from IVD-induced damage of dorsal root ganglions (DRG) and from nociceptive stimulation of DRGs by molecules released from the degenerated IVD (7–9). Indeed, severity of DDD correlates with increased expression of inflammation mediators/markers in the IVD tissue (10), including interleukin-1 beta (IL-1β) (11), tumor necrosis factor-alpha (TNF-α) (12), interleukin-6 (IL-6), and interleukin-8 (IL-8) (13). However, it is still not completely clear how inflammation arises in the IVD and which cellular compartments or molecular pathways are involved in this process.

The endoplasmic reticulum (ER) is a cellular compartment responsible for protein synthesis, signaling, and intracellular calcium homeostasis. Recently, dysregulated ER function has emerged as a possible modulator of/contributor to inflammation in age-related pathologies, such as neurodegenerative diseases, type 2 diabetes, and rheumatoid arthritis (14–16). Dysregulation of ER function, the so-called ER stress, occurs when misfolded and/or unfolded proteins accumulate in the ER. ER stress upregulates chaperone glucose-regulated protein (GRP78, also HSPA5, and Bip), which then triggers unfolded protein response (UPR or ER stress response) to correct protein synthesis by increasing protein-folding capacity, decreasing protein translation rate, and degrading unfolded and misfolded proteins (17). However, prolonged UPR is commonly not able to resolve ER stress, activating pathways leading to cell death. In aging tissues, the UPR function deteriorates, shifting the balance toward degeneration and apoptosis (18).

Endoplasmic reticulum stress in the IVD can be induced by multiple mechanisms, including biomechanical alterations and non-physiological loading: unbalanced dynamic and static forces in the spine activated ER stress in a rat model (19). Likewise, ER stress was present in a rat model of puncture-induced IVD degeneration, where the UPR was accompanied by increased gene expression of IL-6 (20). The expression of GRP78 and its downstream targets in IVD cells was also found to be regulated by compression loading and low pH (21, 22). In addition, serum starvation and glucose deprivation induced ER stress in rat and goat IVD cells (20, 23). As such, ER stress may have protective, but also detrimental effects on IVD homeostasis. Recently, published rodent studies indicated that ER stress-induced apoptosis contributes to IVD degeneration (19, 20) and that ER stress could correlate with inflammation in human IVD tissue (20). Zhao et al. (19) showed that the instability of the lumbar spine significantly upregulated the expression of ER stress markers [GRP78 and CCAAT-enhancer-binding protein homologous protein (CHOP)] as well as markers of apoptosis (caspase-12 and cytochrome C) in the rat IVD. Moreover, the degree of IVD cell apoptosis correlated with the expression of GRP78 (19). Fujii et al. (20) revealed that the induction of ER stress in rat and human AF cells activated gene expression of TNF-α and IL-6, with protein kinase R-like ER kinase (PERK) and nuclear factor κB (NF-κB) likely being involved (20).

Emerging evidence hence suggests that ER stress is present in human degenerated IVDs and contributes to DDD, possibly through induction of inflammation, but the molecular connections between these processes remain unclear. Therefore, the aim of this study was to analyze the relationship and possible crosstalk between ER stress and inflammation in degenerated human IVDs. Understanding the crosstalk between inflammation and ER stress could help in designing therapies that mitigate the harsh environment in degenerated IVD, and thus provide new options for biological IVD repair.

## Materials and Methods

### Surgical IVD Tissue Study

#### Sample Collection

51 human IVD specimens were obtained with informed consent from 45 patients [mean age = 52 years (age 16–79 years)] undergoing elective spinal surgery for treatment of DDD (23 patients) or disc herniation (DH) (22 patients) in the cervical (*n* = 24) or lumbar (*n* = 27) region. Due to the tissue size, cervical samples were collected as entire discs. Lumbar discs were intraoperatively excised as annulus fibrosus (AF, *n* = 12) and nucleus pulposus (NP, *n* = 15) samples, followed by macroscopic tissue evaluation. Assessment of the disease state was performed using Pfirrmann grading (IVD degeneration) and Modic grading (endplate changes). Detailed patient information is given in Ref. (13). The study was approved through the local ethics committee (Ethics Committee of the Canton Lucerne/Switzerland, #1007).

#### Gene Expression Analysis

RNA extraction and the following steps were performed according to Ref. (13). For the cDNA synthesis, 2 µg of RNA were used in a total volume of 60 µL, using the reverse transcription kit (4374966, Applied Biosystems). For samples with lower yields, the reverse transcription was conducted at reduced concentrations. cDNA (10 ng/well) was mixed with TaqMan Fast Universal PCR Master Mix and TaqMan primers (GRP78, IL-6, IL-8, IL-1β, TNF-α, and GAPDH; Table 1) to quantify gene expression. The obtained Ct values were analyzed by a comparative method (gene of interest relative to GAPDH) and displayed as 2^−dCt^ values.

**Table 1:** Human TaqMan primers used in the study.

	Primer target name	Catalog number
1	TATA box binding protein	Hs00427620_m1
2	Glyceraldehyde 3-phosphate dehydrogenase	Hs02758991_g1
3	Interleukin-6	Hs00174131_m1
4	Interleukin-8	Hs00174103_m1
5	Interleukin-1β	Hs00174097_m1
6	Tumor necrosis factor-α	Hs00174128_m1
7	Cyclooxygenase 2	Hs00153133_m1
8	Glucose-regulated protein 78	Hs00607129_gH
9	Activating transcription factor 4	Hs00909569_g1
10	C/EBP homologous protein	Hs00358796_g1
11	Matrix metalloproteinase 1	Hs00233958_m1
12	Matrix metalloproteinase 3	Hs00233992_m1

### *In Vitro* Cell Culture Study

#### Sample Collection for Primary Cell Culture

Mixed human lumbar IVD tissue was removed from 25 patients during IVD surgeries [mean age = 51 years (age 29–76 years)]. Tissue was enzymatically digested using a mixture of 0.2% collagenase NB4 (17454, Serva, Heidelberg, Germany) and 0.3% dispase II (04942078001, Roche, Basel, Switzerland) for 4–8 h at 37°C. Isolated primary cells were seeded in Dulbecco’s Modified Eagle’s Medium (DMEM/F12, D8437, Sigma, St. Louis, MO, USA) supplemented with 10% fetal calf serum (FCS, F7524, Sigma) and 1% Antibiotics–Antimycotics (A/A) (15240062, Gibco, Carlsbad, CA USA) and sub-cultured up to passage 3 using 1.5× trypsin (15090-046, Gibco) at 37°C with 5% CO_2_. This study was approved by the Kantonale Ethikkommission Zürich EK-16/2005 and patient informed consents were granted.

#### Treatments

Interverterbral disc cells were seeded in 6-well plate (2 × 10^5^ cells/well; gene expression analysis), 12-well plate (1 × 10^5^ cells/well; immunoblotting, MTT), or 96-well plate (0.4 × 10^5^ cells/well; calcium flux assay) and cultured for 18 h. The next day, the cells were serum-starved for 2 h and exposed to the compounds shown in the Table 2 for 1, 3, and/or 24 h in FCS- and A/A-free media. Following the treatments, the cells were analyzed by RT-qPCR (gene expression), MTT assay (metabolic activity) and immunoblotting (protein expression). Cell culture media was used for enzyme-linked immunosorbent assay (ELISA) (protein release) and LDH (cytotoxicity test).

**Table 2:** Chemical compounds and siRNAs used in the cell culture experiments.

	Compound	Concentrations	Catalog number (producer)
1	Thapsigargin	100 and 500 nM	T9033 (Sigma)
2	Interleukin 1β	1, 5, and 10 ng/mL	211-11 (Peprotech)
3	Tumor necrsosis factor-α	1, 5, and 10 ng/mL	315-01A (Peprotech)
4	SB203580	10 µM	S8307 (Sigma)
5	siRNA negative control	5 nM	SI03650318 (Qiagen)
6	siRNA C/EBP homologous protein (CHOP)_1	5 nM	SI00059528 (Qiagen)
7	siRNA CHOP_2	5 nM	SI00059535 (Qiagen)

#### Gene Silencing

Intervertebral disc cells were seeded in 12-well plate (1 × 10^5^ cells/well) in DMEM/F-12 supplemented with 10% FCS and 1% A/A and mixed with siRNA complexes according to the manufacturer’s protocol (Fast-Forward protocol, Flexi Tube system, Qiagen). Briefly, 5 nM siRNAs against CHOP (Table 2) and Hiperfect (HF, 301705, Qiagen) were incubated in Opti-MEM (31985, Gibco) for 7 min to form complexes. The complexes were then added to the cell suspension drop-wise and incubated for 18 h. Untreated cells and cells treated with Hiperfect were used as controls. On the next day, the media was changed to FCS-A/A-free media and incubated for 2 h before Tg was added. After 24 h, the cells and culture media were collected for immunoblot analysis of CHOP and ELISA of released IL-6, IL-8, and prostaglandin E2 (PGE2). As siRNA CHOP_2 did not produce sufficient knockdown, only the data obtained with siRNA_CHOP1 were shown.

#### Gene Expression Analysis

RNA was extracted with the Trizol/chloroform (15596-018, Invitrogen, Carlsbad, CA, USA) according to the manufacturer’s instructions. 1 µg of cDNA was reverse transcribed from RNA using a reverse transcription kit (4374966, Applied Biosystems, Foster City, CA, USA) and mixed with the primers (Table 1) and Fast universal master mix (4352042, Applied Biosystems). Gene expression was examined by qPCR. Data were analyzed by the comparative 2^−ΔΔCt^ method, with TATA box binding protein as housekeeping gene. Results were presented as gene expression relative to control (fold change) or gene expression relative to Tg treatment (%).

#### Immunoblotting

Cells were lysed and mixed with Laemmli buffer (S3401, Sigma). The lysates were boiled (96°C, 5 min) and loaded onto 4–20% SDS-polyacrylamide gels (456-8093, Biorad). Proteins were separated by electrophoresis in a Mini-PROTEAN Tetra cell chamber (Biorad) and transferred to polyvinylidene difluoride membranes (1704156, Biorad). Following transfer, the membranes were blocked in 5% non-fat milk in Tris-buffered saline-Tween (TBS-T) for 1 h at room temperature and primary antibodies (Table 3) were applied overnight at 4°C under gentle shaking. The next day, membranes were washed in 1% non-fat milk in TBS-T (3 × 10 min), incubated with secondary antibodies conjugated to horseradish peroxidase (HRP) for 1 h at room temperature, and then washed again in 1% non-fat milk in TBS-T (3 × 10 min). Tubulin was used as loading control. Visualization was performed by chemiluminescence kit West Dura (34076, Thermo Scientific, Waltham, MA, USA) on ChemiDoc imager (Biorad). The obtained bands were quantified in ImageJ x64 by normalizing to loading control and calculating band density relative to untreated control. Resulting graphs show an average of three independent donors.

**Table 3:** Primary and secondary antibodies used for immunoblotting.

	Antibody	Dilution	Cat. no., producer
1	α-Tubulin	1:1,000	2144, cell signaling
2	p38 MAPK	1:1,000	9212, cell signaling
3	Phospho-p38 MAPK	1:1,000	9211, cell signaling
4	Phospho-JNK	1:1,000	9251, cell signaling
5	Phospho-nuclear factor κB (NF-κB) p65	1:1,000	3033, cell signaling
6	Cyclooxygenase (COX-2)	1:1,000	12282, cell signaling
7	Inhibitor of NF-κB alpha (IKB-α)	1:1,000	9242, cell signaling
8	Protein kinase R-like endoplasmic reticulum kinase (PERK)	1:1,000	5683, cell signaling
9	C/EBP homologous protein (CHOP)	1:1,000	2895, cell signaling
10	IRE1α	1:1,000	3294, cell signaling
11	Glucose-regulated protein (GRP78)	1:1,000	3177, cell signaling
12	Anti-mouse IgG, horseradish peroxidase (HRP)-linked	1:3,000	7076, cell signaling
13	Anti-rabbit IgG, HRP-linked	1:3,000	7074, cell signaling

#### Enzyme-Linked Immunosorbent Assay

Inflamed IVD cells typically release pro-inflammatory cytokines (24, 25). IL-6 and IL-8 secretion was measured by ELISA according to the producer’s protocols (BD OptEIA™ Human IL-6 ELISA set, 555220; Human IL-8 ELISA set, 555244; and Reagent set B, 550534, BD Biosciences, San Jose, CA, USA). Briefly, 96-well plate was coated by kit capture antibody at 4°C overnight, washed, and blocked. Samples and standards were pipetted into the wells in duplicates and the plates were incubated for 2 h at room temperature. The plates were washed and kit detection antibodies and solutions were applied. After the signal was developed, absorbance was measured at 450 nm with 570 nm correction. Total IL-6 and IL-8 release in culture media was plotted (pg/mL). PGE2 is a product of COX-2-mediated enzymatic transformation of arachidonic acid. PGE2 release in culture media was measured by PGE2 ELISA (KGE004B, RD systems) according to the manufacturer’s recommendations. Results are presented as protein release (pg/mL) or the release relative to Tg treatment (%).

#### Cytotoxicity Assay (LDH)

To verify whether performed treatments influenced cell viability, Cytotoxicity Assay Kit (88954, Pierce) was used to measure lactate dehydrogenase (LDH) release into the culture media according to the manufacturer’s recommendations. Results were displayed as cytotoxicity relative to control (fold change).

#### Metabolic Activity Assay (MTT)

To verify whether performed treatments influenced cell metabolic activity, MTT (3-[4,5-dimethylthiazol-2-yl]-2,5-diphenyl tetrazolium bromide, M5655, Sigma) was used. Following seeding and treatments, fresh MTT solution (0.5 mg/mL) was added and kept for 3 h at 37°C. MTT was discarded, cells were lysed in DMSO (D8418, Sigma), and the absorbance was measured at 565 nm. Metabolic activity was calculated relative to the untreated control (100%).

#### Calcium Flux Assay

Unbalanced intracellular calcium levels can initiate ER stress. Intracellular calcium [Ca^2+^]_i_ was analyzed by Fura-2-AM solution (Fura-2 QBT™ Calcium Kit, component A, Molecular Devises, CA, USA) according to the manufacturers’ recommendations. Briefly, IVD cells were seeded in 96-well plate and incubated for 18 h. The next day, the media was changed to the phenol red-free media (11039, Gibco) supplemented with Fura-2-AM solution and cells were incubated for 1 h. Fura-2 fluorescence was recorded using a plate reader (Infinite M200pro TECAN, Switzerland) at excitation wavelengths of 340 and 380 nm and an emission wavelength of 510 nm. After 5 cycles, the cells were exposed to putative calcium flux activators IL-1β (10 ng/mL), TNF-α (10 ng/mL), and Tg (100 and 500 nM), with 1 µM ionomycin (I3909, Sigma) as positive control. Fura-2 fluorescence continued to be recorded for up to 15 min. The assay was performed in triplicates. The data were presented as the ratio of 340/380 signal.

### Statistical Methods

Data consistency was checked and data were screened for outliers by using quantile plots and normality using Kolmogorov–Smirnov test. Due to the given distributions, generalized linear models were not applicable and, hence, Wilcoxon-matched pairs test, Mann–Whitney *U* test and Spearman correlation was used to analyze continuously distributed data. One-factorial ANOVA with Tukey’s *post hoc* test was used to test means among different groups. All reported tests were two sided, and *p*-values <0.05 were considered to be statistically significant. All statistical analyses in this report were performed using STATISTICA 13 (Hill, T. & Lewicki, P. Statistics: methods and applications. StatSoft, Tulsa, OK, USA) GraphPad Prism 7 and PASW 22 (IBM SPSS Statistics for Windows, Version 21.0., Armonk, NY, USA) and StatXact 10 (Cytel Software 2013, Cambridge MA, USA).

## Results

### The Expression of GRP78 and Inflammatory Cytokines in Degenerated IVDs

**Figure 1:**
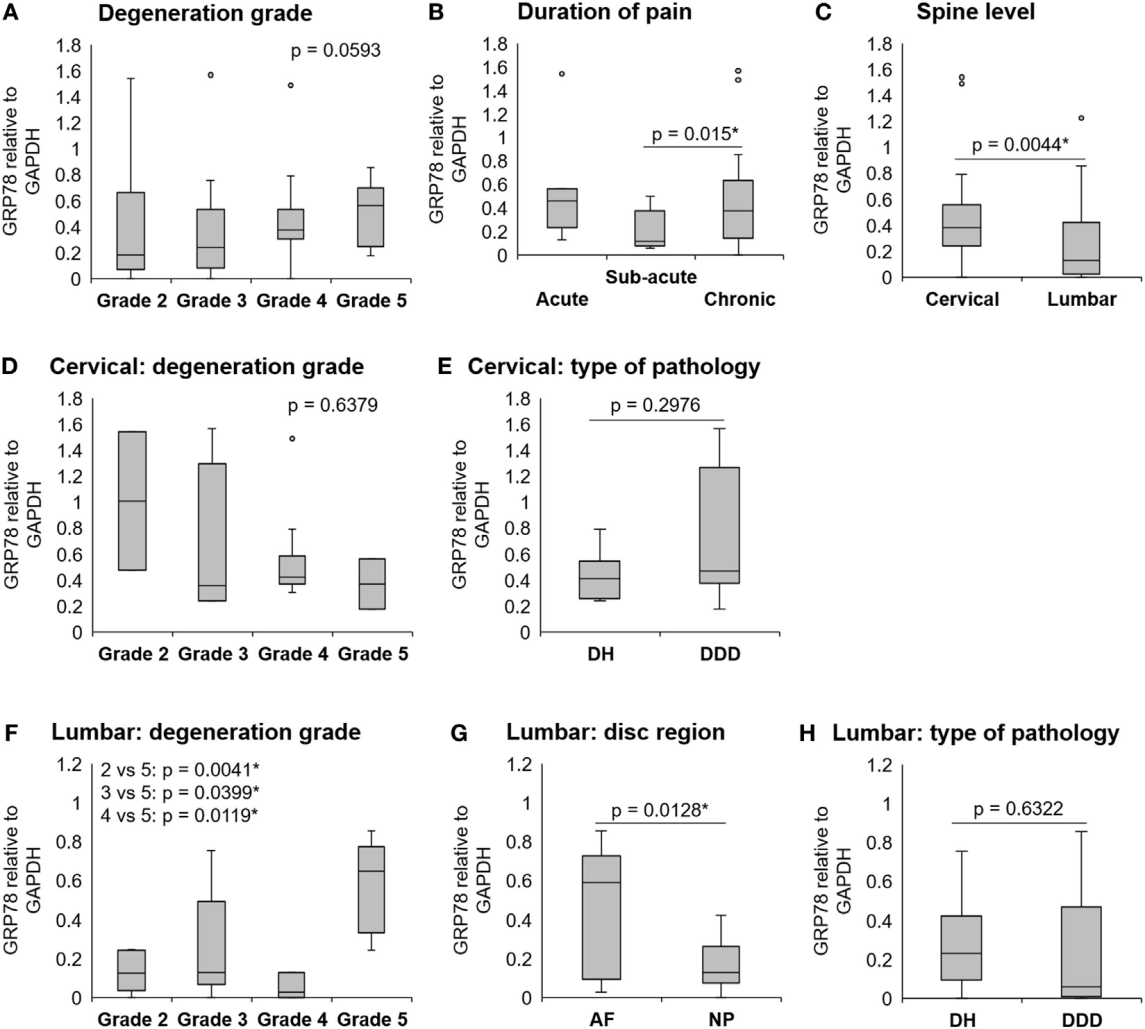
The expression of GRP78 in human degenerated intervertebral discs (IVDs). IVDs were collected from donors during spinal surgeries. Gene expression of GRP78 in cervical and lumbar IVDs (*n* = 51 donors) **(A)** tended to increase with degeneration grade (*p* = 0.0593) and significantly increased with **(B)** duration of pain (*p* = 0.015) and **(C)** in cervical IVDs, when compared with lumbar IVDs (*p* = 0.0044). **(D)** Gene expression of GRP78 in cervical IVDs (*n* = 20 donors) was not significantly different between grades and **(E)** type of pathology. **(F)** Gene expression of GRP78 in lumbar IVDs (*n* = 25 donors) increased with degeneration grade (2 vs 3 *p* = 0.0019, 2 vs 4 *p* = 0.0399, and 2 vs 5 *p* = 0.0041). **(G)** A difference in GRP78 expression was found between the disc regions [*n* = 12 (AF), 15 (NP), *p* = 0.0128], but not **(H)** between different pathologies. Calculated as 2^−ΔCt^ values (relative to GAPDH). Abbreviations: DDD, degenerative disc disease; DH, disc herniation; AF, annulus fibrosus; NP, nucleus pulposus.

Gene expression of ER stress marker GRP78 in human IVD samples in relation to the IVD degeneration grade [grades 2–5 (26)], duration of pain, spine level (cervical or lumbar), type of pathology (DDD or DH), and disc region (NP or AF) are illustrated (Figure 1). GRP78 was expressed in 20 out of 24 cervical samples, 11 out of 12 lumbar AF samples, and 14 out of 15 lumbar NP samples. GRP78 expression in cervical and lumbar IVDs together showed a tendency to increase with the degeneration grade (*p* = 0.0593; Figure 1A) and association with the duration of the pain symptoms (*p* = 0.015, sub-acute vs chronic; Figure 1B), with high variability in the acute group. The expression of GRP78 was significantly lower in lumbar IVDs, when compared to cervical IVDs (*p* = 0.0044; Figure 1C). Due to the possible morphological and biomechanical differences between cervical and lumbar IVDs, the expression of GRP78 was displayed separately in cervical and lumbar spine in relation to the degeneration grade and type of pathology. Cervical IVDs showed no association between the expression of GRP78 and degeneration grade (Figure 1D) and the type of pathology (Figure 1E). In lumbar IVDs, the expression of GRP78 significantly increased with increasing degeneration grade (2 vs 3 *p* = 0.0019, 2 vs 4 *p* = 0.0399, and 2 vs 5 *p* = 0.0041; Figure 1F). However, caution must be taken when interpreting these data, as grades 2 and 5 (cervical) contain only two samples and grade 4 (lumbar) contains only three samples. GRP78 was expressed more in AF than NP (*p* = 0.0128) (Figure 1G). The expression of GRP78 was not significantly different between DDD and DH (Figure 1H). The expression of GRP78 in NP and AF samples in relation to degeneration grade and type of pathology is shown in the Figure S1 in Supplementary Material.

The expression of inflammation markers, such as IL-1β, TNF-α, IL-6, and IL-8 in these samples was measured as well (13). Interestingly, the expression of GRP78 positively correlated with IL-6 (*r* = 0.63, *p* < 0.0001) and IL-8 (*r* = 0.47, *p* = 0.0001), while no correlation between the expression of GRP78 and IL-1β or TNF-α was found (*p* = 0.4641, *p* = 0.1709, respectively) (Table 4). To confirm the presence of ER stress in IVD tissue, activating transcription factor (ATF4) was measured as another ER stress marker (*n* = 13). GRP78 positively correlated with ATF4 (*r* = 0.62, *p* = 0.0105). The expression of ATF4 positively correlated with the inflammation markers IL-6 (*r* = 0.72, *p* = 0.0021) and IL-8 (*r* = 0.63, *p* = 0.0110) (Table 4).

**Table 4:** The expression of GRP78 is positively correlated with IL-6 and IL-8, but not with IL-1β and TNF-α. The expression of ATF4 is positively correlated with GRP78, IL-6, and IL-8 (calculated by Spearman correlation test).

Pair of variables	Sample size	Correlation *r*	*p*-value
GRP78 and IL-6	45	0.63	<0.0001
GRP78 and IL-8	45	0.47	0.0010
GRP78 and IL-1β	45	0.11	0.4641
GRP78 and TNF-α	45	0.21	0.1708
ATF4 and GRP78	13	0.62	0.0105
ATF4 and IL-6	13	0.72	0.0021
ATF4 and IL-8	13	0.63	0.0110

### The expression of GRP78 in IVD cells Is Associated With Calcium Depletion and ER Stress

**Figure 2:**
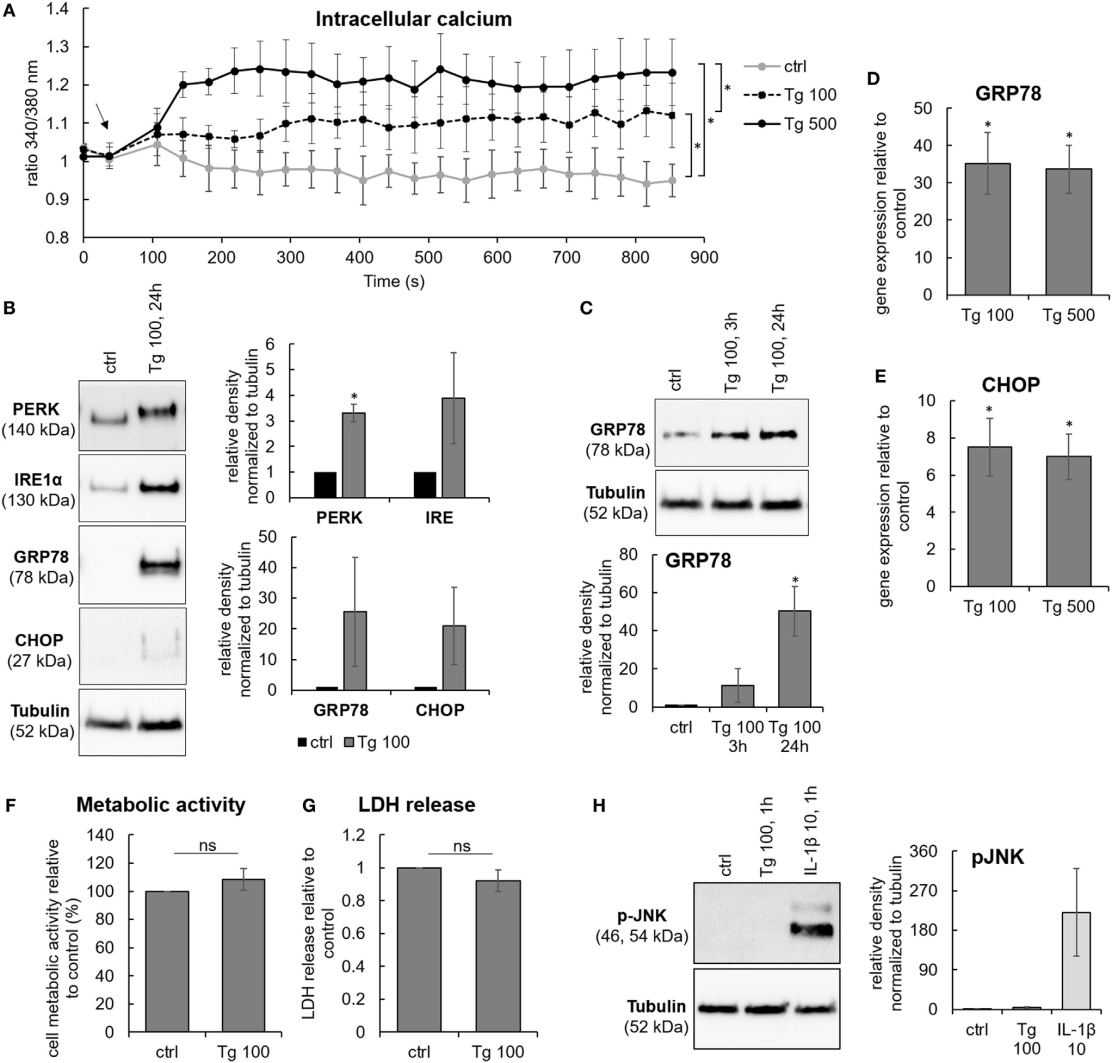
Sub-lethal endoplasmic reticulum (ER) stress induction by thapsigargin in intervertebral disc (IVD) cells. IVD cells were isolated from degenerated lumbar IVDs and treated with 100 and 500 nM thapsigargin (Tg). Treatment with Tg **(A)** caused steady calcium flux (*n* = 3 donors; the arrow indicates the time point when Tg was added), **(B,C)** induced the expression of ER stress-associated proteins PERK, IRE, GRP78, and C/EBP homologous protein (CHOP) (*n* = 3 donors), and **(D,E)** activated gene expression of GRP78 and CHOP (*n* = 7 donors; relative to control = set at 1). **(F–H)** 100 nM Tg did not influence cell metabolic activity (*n* = 3 donors; relative to control = set at 100) and viability (*n* = 13 donors; relative to control = set at 1), nor activated JNK (*n* = 4 donors). IL-1β (10 ng/mL) was used as positive control for immunoblotting. Data are presented as mean ± SEM, *p < 0.05 (ANOVA with Tukey *post hoc* test).

Due to the possible association between ER stress and degeneration grade in the lumbar region, cells isolated from lumbar IVDs were used in the following cell culture study. It has been shown that IVD cells can respond to stress by increasing the concentration of [Ca^2+^]_i_ (27). Therefore, thapsigargin (Tg), an inhibitor of the sarco/ER Ca^2+^ ATPase, was used to chemically induce ER stress in IVD cells, as it raises [Ca^2+^]_i_ by blocking the ability of the cell to pump calcium into the ER (28). IVD cells were treated with 100 and 500 nM Tg for 1, 3, or 24 h. Tg treatment elevated [Ca^2+^]_i_ (Figure 2A) and increased the expression of ER stress markers, such as PERK, IRE1α, GRP78, and CHOP on the protein level (Figures 2B,C) and gene level (Figures 2D,E). The effects of 100 nM Tg on cell death were also tested. Tg did not significantly reduce cellular metabolic activity nor induce cell death (Figures 2F,G). ER stress-induced JNK activation, known to influence cell death through the regulation of BCL2 family proteins (29), was not observed (Figure 2H). Therefore, Tg was used further to mimic sub-lethal ER stress in IVD cells. *P*-values are shown in the Table S1 in Supplementary Material.

### ER Stress Activates the Expression of IL-6 Through p38 MAPK and CHOP

**Figure 3:**
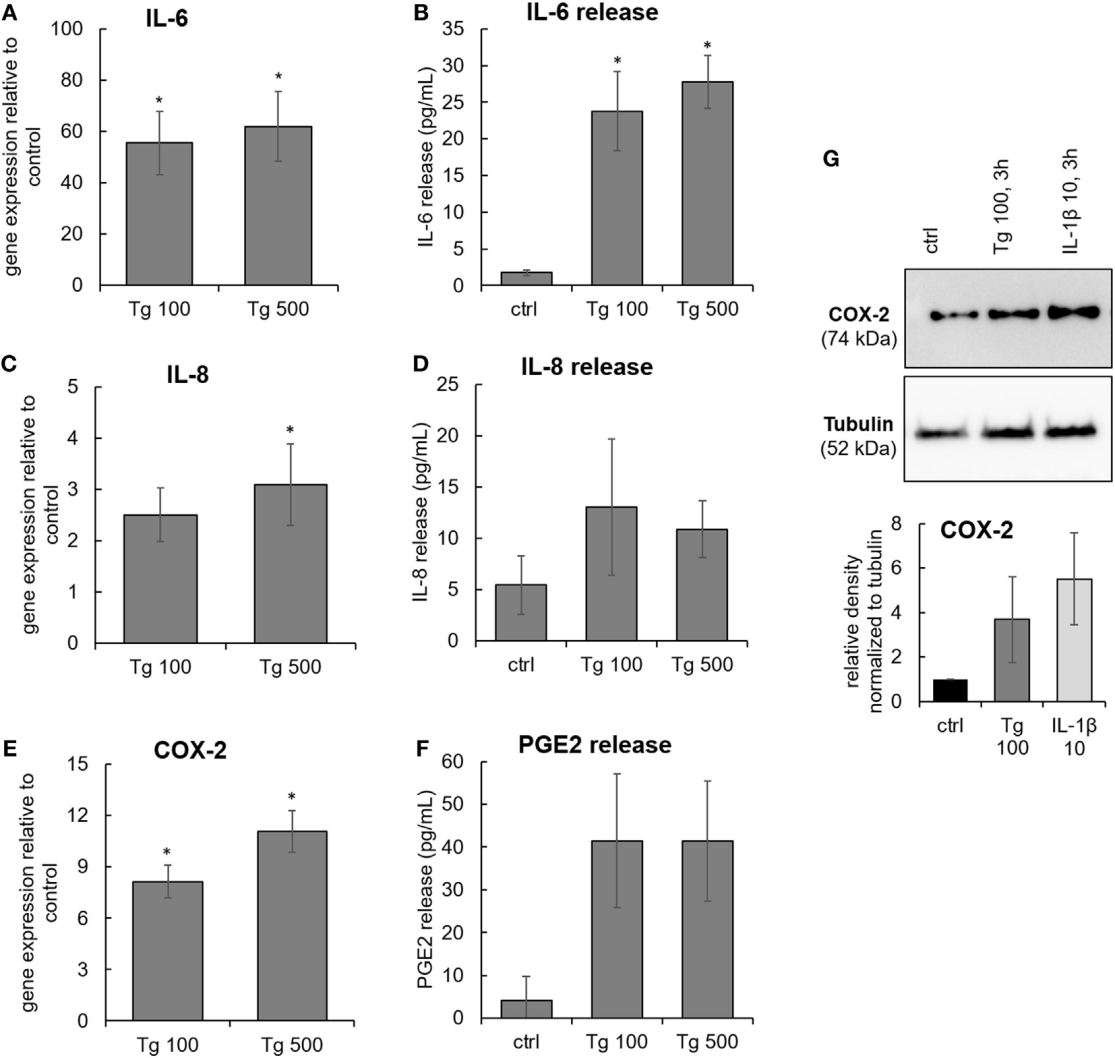
The expression of IL-6, IL-8, and COX-2 in endoplasmic reticulum stress. Cells were isolated from degenerated lumbar intervertebral discs (IVDs) and treated with 100 and 500 nM thapsigargin (Tg). **(A,C,E)** Treatment with Tg significantly induced gene expression of IL-6, IL-8, and COX-2 (*n* = 7 donors, relative to control = set at 1) and **(B,D,F)** secretion of IL-6, but not IL-8 and PGE2 (*n* = 7 donors). **(G)** Tg treatment elevated the protein expression of COX-2 (*n* = 4 donors). IL-1β (10 ng/mL) was used as positive control for immunoblotting. Data are presented as mean ± SEM, *p < 0.05 (ANOVA with Tukey *post hoc* test).

The expression of inflammation-associated genes and proteins was tested in IVD cells treated with 100 and 500 nM Tg for 24 h. ER stress caused by Tg significantly induced gene expression of IL-6, IL-8, and COX-2 (Figures 3A,C,E). ER stress also significantly activated protein release of IL-6 (Figure 3B), indicating the involvement of ER stress in inflammatory responses of IVD cells. The release of IL-8 in the Tg group was not significantly elevated (Figure 3D), likely due to the modest increase in gene expression. Increased expression of COX-2 protein in Tg-treated cells was observed (Figure 3G). The protein release of PGE2, a downstream product COX-2, tended to increase in the Tg group, but not significantly (Figure 3F). It should be noted that PGE2 release was detectable only in one control sample and that the PGE2 release could be over/under estimated due to the kit detection limit (40 pg/mL). Therefore, the presented PGE2 data should be interpreted with caution. Tg did not influence the expression of matrix metalloproteinases 1 and 13, while IL-1β and TNF-α were expressed neither in controls, nor in Tg-treated samples (Ct values higher than 40, data not shown). Inter-donor differences in Tg-induced protein expression and release were detected. *P*-values of all tests are shown in the Table S1 in Supplementary Material.

**Figure 4:**
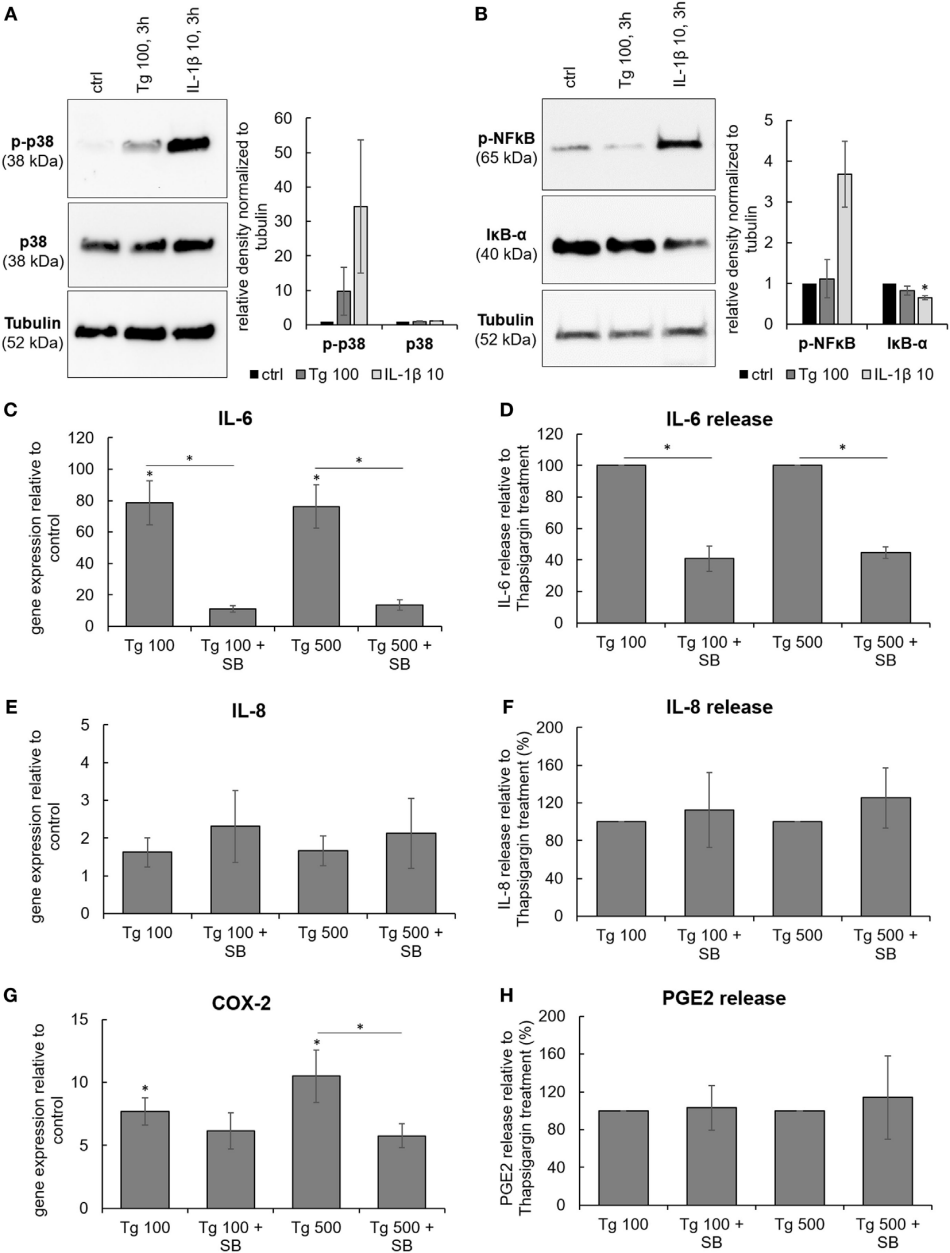
The involvement of p38 MAPK in the expression and release of IL-6, IL-8, and PGE2. Cells were isolated from degenerated lumbar intervertebral discs and treated with 100 nM thapsigargin (Tg) alone or in combination with 10 µM small molecule inhibitor of p38 SB203580 (SB). **(A)** 3 h treatment with Tg induced phosphorylation of p38 MAPK (*n* = 4 donors). **(B)** NF-κB pathway (tested by phosphorylation of NF-κB and simultaneous degradation of IKB-α) was not activated by 3 h treatment with Tg (*n* = 4 donors). **(C,E,G)** Combination of Tg and SB reduced gene expression of IL-6, and COX2, but not IL-8 (*n* = 4 donors). **(D,F,H)** SB reduced protein release of IL-6, but not IL-8 and PGE2 (*n* = 4 donors). IL-1β (10 ng/mL) was used as a positive control for immunoblotting. Data are presented as mean ± SEM, *p < 0.05 (ANOVA with Tukey *post hoc* test).

The functional crosstalk between ER stress and inflammatory responses was examined by testing activation of typical mediators involved in IVD inflammation, namely p38 mitogen-activated protein kinase (p38 MAPK) and NF-κB. A common inducer of these pathways, IL-1β (10 ng/mL), was used as a positive control. 3 h treatment with 100 nM Tg stimulated the phosphorylation of p38 MAPK (Figure 4A). The NF-κB pathway, induction of which manifests by phosphorylation of NF-κB (p65) and degradation of its inhibitor IκB-α, was not activated by 100 nM Tg (Figure 4B).

To test the functional involvement of p38 in Tg-induced gene and protein expression of IL-6, IL-8, and COX-2, the small molecule inhibitor of p38 MAPK SB203580 (SB; 10 µM) was used. Inhibition of p38 significantly reduced gene expression of IL-6 and COX-2, while the expression of IL-8 remained unchanged (Figures 4C,E,G). In addition, the release of IL-6 into the culture media was significantly inhibited by SB, indicating functional involvement of p38 in ER stress-induced inflammatory responses of the IVD. SB did not influence the release of IL-8 and PGE2 (Figures 4D,F,H). Cell viability (tested by LDH assay) in all treatment groups remained comparable to control (Figure S2 in Supplementary Material).

**Figure 5:**
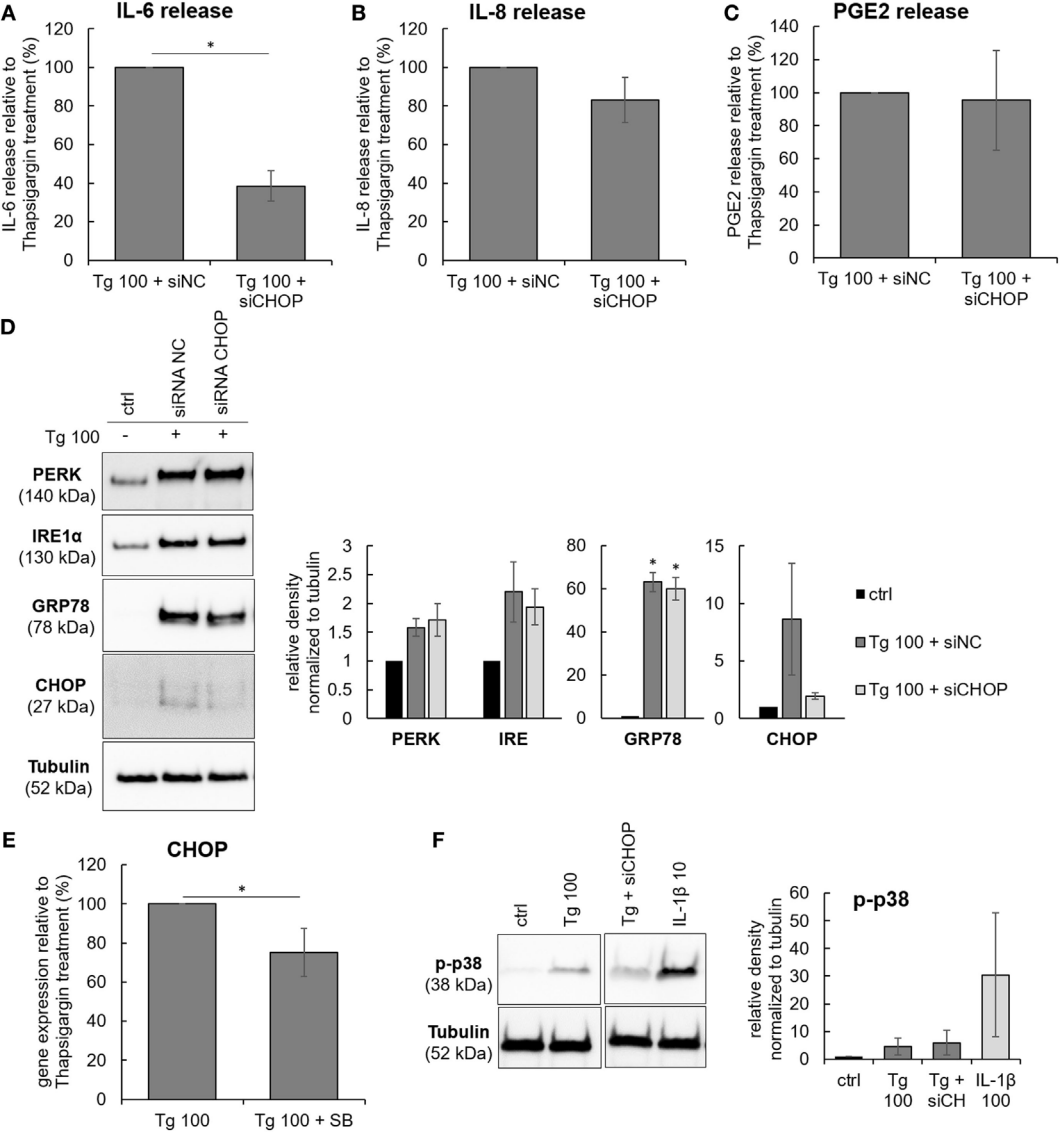
The effects of C/EBP homologous protein (CHOP) knockdown on the protein release of IL-6, IL-8, and PGE2. Cells were isolated from degenerated lumbar intervertebral discs (IVDs) and treated with 100 nM thapsigargin (Tg) in combination with 5 nM negative control siRNA (siRNA NC, scrambled) or 5 nM siRNA against CHOP (siCHOP). **(A–C)** CHOP knockdown reduced the release of IL-6, but not IL-8 and PGE2 (*n* = 3 donors). **(D)** Tg-activated endoplasmic reticulum stress, as shown by expression of GRP78, PERK, IRE1α, and their downstream effector CHOP. siRNA produced partial knockdown of CHOP gene. **(E)** p38 inhibition partially reduced gene expression of CHOP in Tg-treated cells (*n* = 4 donors). **(F)** CHOP knockdown did not reduce p38 phosphorylation by Tg (*n* = 3 donors). Data are presented as mean ± SEM, *p < 0.05 (ANOVA with Tukey *post hoc* test).

A putative downstream target of p38 is CHOP (or GADD153), a transcription factor that mediates ER stress-induced autophagy, apoptosis, or inflammation (30). The involvement of CHOP in the observed effects of Tg was tested by CHOP siRNA knockdown. The CHOP knockdown (Figure 5D) reduced the release of IL-6, indicating the participation of CHOP in ER stress-induced inflammatory responses of IVD cells. CHOP knockdown did not influence the release of IL-8 and PGE2 (Figures 5A–C). The transfection reagent Hiperfect (HF) did not influence gene expression of CHOP and IL-6 release (Figure S3 in Supplementary Material). Cell viability in all treatment groups remained similar to control (tested by LDH) (Figure S2 in Supplementary Material). The relationship between p38 and CHOP was evaluated in Tg-treated cells by gene expression analysis of CHOP with and without SB as well as by activation of p38 in the presence and absence of siRNA against CHOP. p38 inhibition partially reduced gene expression of CHOP (Figure 5E), while CHOP knockdown did not seem to reduce p38 phosphorylation (Figure 5F). However, caution must be taken when interpreting quantified immunoblotting results, as non-significant results may arise from disease-related inter-donor variability.

### IL-1β Activates the Expression of GRP78, but Does Not Significantly Influence Calcium Flux and the Expression of CHOP

**Figure 6:**
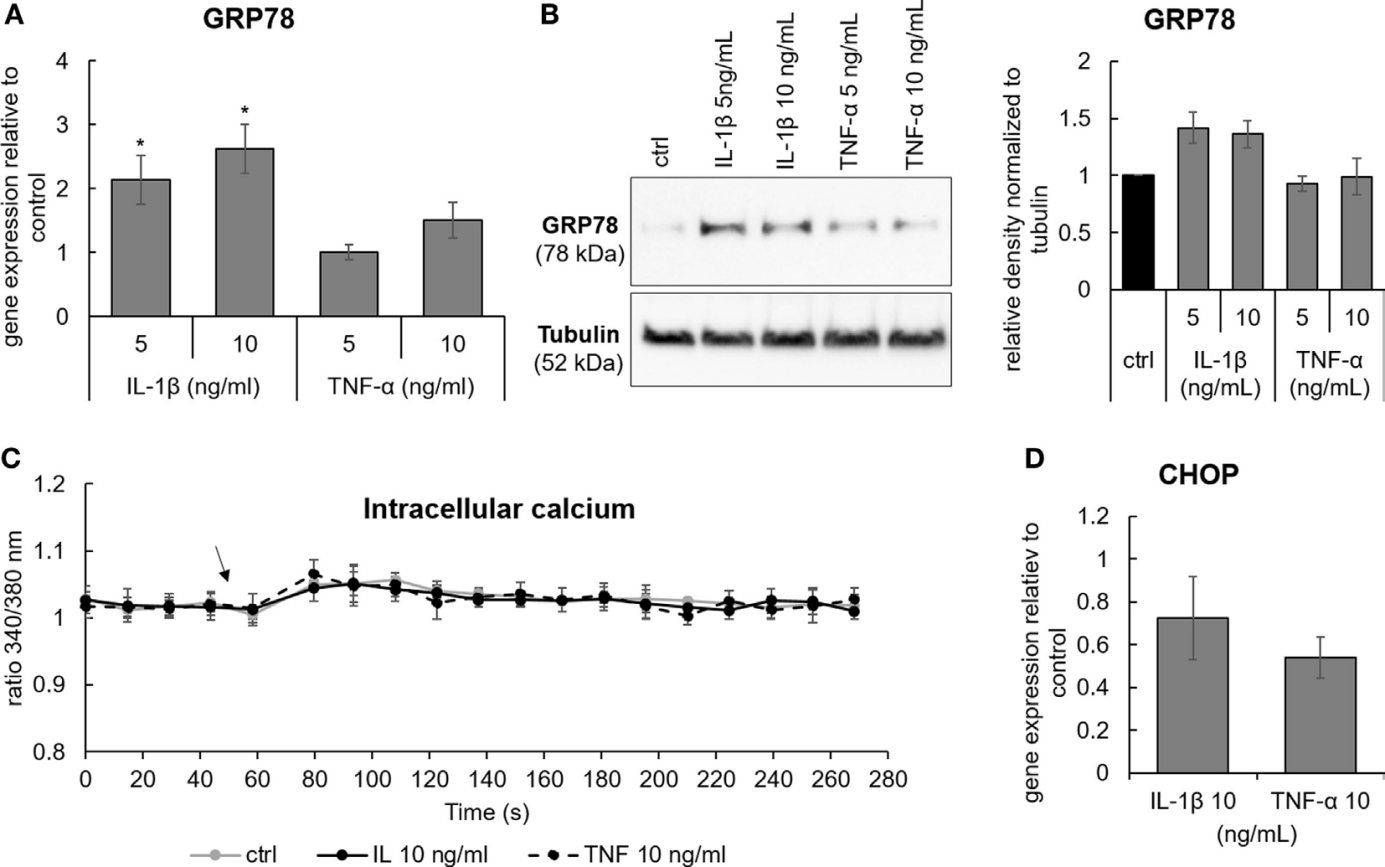
The effects of pro-inflammatory cytokines Il-1β and TNF-α on the expression of GRP78, CCAAT-enhancer-binding protein homologous protein (CHOP), and calcium flux. Cells were isolated from degenerated intervertebral discs and treated with 5 and 10 ng/mL of IL-1β and TNF-α. **(A,B)** IL-1β, but not TNF-α, activated gene and protein expression of GRP78 [*n* = 4 donors (gene), *n* = 3 donors (protein)]. **(C)** Activation of [Ca^2+^]_i_ flux by IL-1β and TNF-α was not found (*n* = 3 donors; the arrow indicates the time point when the cytokines were added). **(D)** Neither IL-1β nor TNF-α activated gene expression of CHOP (*n* = 4 donors). Data are presented as mean ± SEM, *p < 0.05 (ANOVA with Tukey *post hoc* test).

Various events involved in IVD degeneration can possibly activate ER stress either directly or through disturbed [Ca^2+^]_i_ homeostasis. Pro-inflammatory cytokines IL-1β and TNF-α are common inducers of inflammatory responses in the IVD. As an example, IL-1β was shown to activate p38-mediated expression of IL-6, COX-2, and PGE2 in Ref. (31) and in Figure S4 in Supplementary Material. Therefore, the next step was to test the effects of IL-1β and TNF-α on [Ca^2+^]_i_ and ER stress. It was found that 5 and 10 ng/mL IL-1β, but not 5 and 10 ng/mL TNF-α, slightly induced gene and protein expression of GRP78 (Figures 6A,B). Both cytokines neither activated [Ca^2+^]_i_ flux (Figure 6C), nor the expression of CHOP (Figure 6D), suggesting that IL-1β and TNF-α alone are not able to induce ER stress.

## Discussion

Cytokines including IL-6, IL-8, or TNF-α are significantly elevated in degenerated human IVDs (32) and contribute to the inflammatory responses (25) and neuronal sensitization during LBP (33). However, the sources of inflammation in the IVD are still not fully identified. Over the past years, the participation of the ER in IVD homeostasis has been increasingly studied (20), but the role of ER stress in the pathogenesis of DDD still remains unclear. This study aimed to investigate the involvement of ER stress in inflammatory responses of the IVD and to elucidate the molecular crosstalk between these processes.

The study showed an association between ER stress and degeneration grade in lumbar, but not cervical IVDs, which could arise from differences in morphology and biomechanics between these regions. In lumbar IVDs, GRP78 was significantly more expressed in the AF, which could be explained by its association with stretch-induced behavior (34).

To our knowledge, this is the first study that tested and found a correlation between gene expression of IL-6 and ER stress markers in human surgical IVD tissue. The complementary cell culture tests confirmed that ER stress regulates the expression and release of IL-6 by IVD cells, supporting the observations from the patient’s IVDs. Previously, it has been shown that ER stress is accompanied by increased gene expression of IL-6 in a rat model of puncture-induced IVD degeneration (20). ER stress also activated the expression of COX-2, an enzyme involved in the synthesis of PGE2 and clinically relevant target of nonsteroidal anti-inflammatory drugs. Although our data on PGE2 release did not correspond to the COX-2 gene expression data, it should be noted that the measured values were close to the lower detection limit of the used ELSIA (40 pg/mL), and thus this data should be interpreted with caution (for a comparison, the lower detection limits of IL-6 and IL-8 ELISAs were 4 pg/mL).

Common inducers of inflammatory responses in the IVD, IL-1β and TNF-α, were previously shown to activate ER stress in IVD cells (35): 10 ng/mL TNF-α upregulated ER stress markers and initiated UPR in rat IVD cells (36), while 75 ng/mL IL-1β activated gene and protein expression of ER stress markers (including GRP78 and CHOP) in human primary IVD cells (37). However, in our study, IL-1β and TNF-α did not induce a prolonged ER stress response. Although 10 ng/mL IL-1β enhanced the expression of GRP78, it did not activate calcium flux and gene expression of CHOP. The data suggest that IL-1β and TNF-α alone are likely not able to induce ER stress in degenerated human IVDs *in vivo*, where their concentrations are typically lower than in our study (32). This could explain the lack of correlation between IL-1β, TNF-α, and GRP78 in surgical IVD samples. Importantly, IL-6 release in human IVD cells could be initiated without participation of IL-1β and TNF-α, as a direct consequence of ER stress.

**Figure 7:**
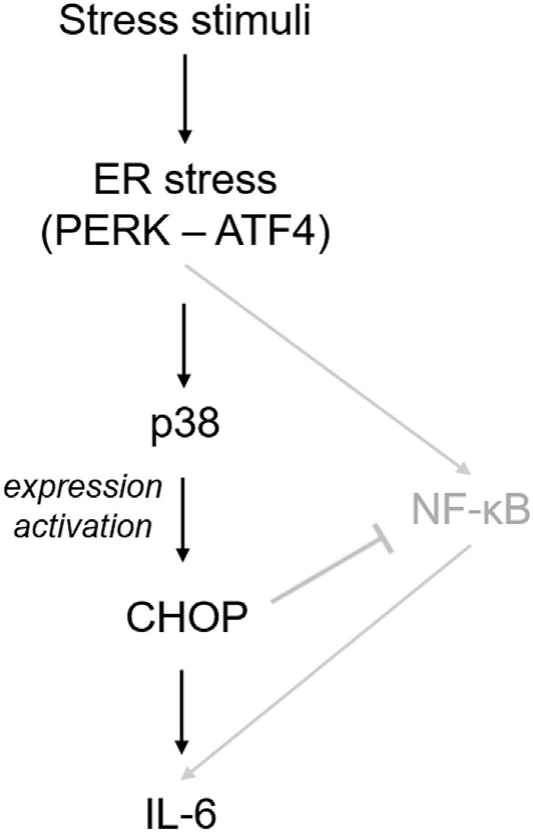
Suggested mechanism of endoplasmic reticulum (ER) stress-induced inflammatory responses in intervertebral disc (IVD) cells. Stress stimuli (such as biomechanical alterations or low pH) can cause dysregulation of calcium homeostasis and sub-lethal ER stress. This in turn activates p38, which induces the expression and phosphorylation of C/EBP homologous protein (CHOP) and subsequent IL-6 release. In its initial phase, ER stress response may help IVD cells to cope with non-physiological stimuli, while severe ER stress may promote inflammation and apoptosis. A previous IVD-related study (20) showed that ER stress activates IL-6 release through NF-κB, which is negatively regulated by CHOP (shown in gray).

Not only cytokines, but also other factors contribute to the harsh microenvironment in degenerated IVDs. Factors like biomechanical alterations, low pH, and glucose deprivation were shown to induce ER stress in IVD tissue and cells (20–23). Our data suggest that ER stress can activate IL-6 release through p38 and CHOP, as shown in the scheme (Figure 7). Prolonged p38 activation is generally associated with cellular stress and induces inflammatory responses (31), cell cycle arrest (38), or apoptosis (39). For example, p38 was shown to promote ER stress-induced apoptosis in fibroblasts (40) and ER stress-induced inflammation in macrophages (41). Importantly, p38 can phosphorylate CHOP and this phosphorylation is required for CHOP activation (42, 43). In addition, p38 can activate the transcription of CHOP through ATF6 (44). In our study, p38 inhibition partially reduced gene expression of CHOP, while CHOP knockdown did not reduce p38 phosphorylation. Therefore, we suggest that the observed ER stress-induced IL-6 release may be partially dependent on transcriptional regulation of CHOP by p38.

In the previous study on rat AF cells, ER stress activated gene expression of inflammatory cytokines through the NF-κB pathway (20). We found no JNK and NF-κB activation at the 1 and 3 h time point, which were selected to test prolonged activation of inflammatory mediators commonly associated with ER stress response (45, 46). Possible transient activation of JNK and/or NF-κB at shorter time points (e.g., 15 min) cannot be excluded. It has been shown that NF-κB-induced IL-6 release in the IVD can be negatively regulated by CHOP (20), suggesting yet unexplored crosstalks between ER stress and inflammation may exist (Figure 7). Severe ER stress is known to activate cell death. In previous ER stress-related studies, active JNK-induced caspase-12-mediated apoptosis (47, 48), while ER stress-induced activation of another MAP kinase ERK (not tested in our study) had pro-survival effects (49).

The activation of CHOP has traditionally been considered pro-apoptotic (50); however, in our study the mild induction of CHOP was not associated with cell death. Recent literature suggests that CHOP plays a role in the pathogenesis of inflammation (other cell types) (30, 51), could thus constitute a link between ER stress and inflammatory responses in the IVD. As an example, CHOP was shown to be involved in the release of IL-6 in macrophages during atherosclerosis (41) and generation of reactive oxygen species (ROS) and ROS-induced damage in diabetes (52, 53). The mechanistic details on CHOP involvement in IL-6 release should be investigated further, preferably in IVD cells with naturally induced ER stress (e.g., by low pH or starvation).

Tissues and primary cells were isolated from randomized donors with different demographics (e.g., age and associated pathologies). Commonly, natural variability in such sample group leads to inter-donor differences in protein activation and release, reducing potential statistical significance of observed effects. Although not all statistically significant, our data suggest that ER stress could indeed participate in inflammatory responses of the IVD through p38 and CHOP. Statistically significant association between ER stress and IVD degeneration was confirmed previously in animal models, which do not suffer from non-uniformity arising from natural variability (19).

It is known that the harsh microenvironment in degenerated IVD can hinder biological regenerative therapies (54). Therapeutic modulation of ER stress could possibly contribute to mitigate these effects. ER stress-inflammation crosstalk could be therapeutically targeted on several levels. [Ca^2+^]_i_ dysregulation and/or protein misfolding could be targeted by molecular chaperones, compounds that prevent aggregation and promote ER function, a decrease of which is typical for degenerative disorders (55). To date, the beneficial effects of some compounds modulating ER stress responses (e.g., Tauroursodeoxycholic acid) have been shown in preclinical studies for neurodegenerative, cardiovascular, and metabolic disorders (55–57). ER stress effectors, such as the transcription factor CHOP, could be modulated, e.g., by gene editing using clustered regularly interspaced short palindromic repeats (CRISPR)/CRISPR-associated protein 9 techniques (knock-out, knock-down) (58). It should not be forgotten that ER stress in its initial stages has protective effects toward restoring cellular homeostasis. Healthy cells are able to resolve ER stress by increased expression of molecular chaperones and UPR genes, and thus inhibition of UPR molecules (e.g., GRP78, PERK, and IRE) may not be desired at this stage. On the other hand, chronic ER stress has detrimental consequences that inhibition of ER stress effectors (e.g., CHOP) could help to reduce.

## Conclusion

This study is one of the first to describe an active role of ER stress in inflammatory responses of human IVD. More studies will follow to elucidate the importance of related molecular mechanisms as therapeutic targets to mitigate the harsh microenvironment of degenerated IVD.

## Ethics Statement

51 human IVD specimens were obtained with informed consent from 45 patients [mean age = 52 years (age 16–79 years)] undergoing elective spinal surgery for treatment of degenerative disc disease (23 patients) or disc herniation (22 patients) in the cervical (*n* = 24) or lumbar (*n* = 21) region. The study was approved through the local ethics committee (Ethics Committee of the Canton Lucerne/Switzerland, #1007). Human IVD tissue for the cell tests was removed from 25 patients during IVD surgeries [mean age = 51 years (age 29–76 years)]. The study was approved by the Kantonale Ethikkommission Zürich EK-16/2005. Patient informed consents were granted.

## Author Contributions

OK planed the study, drafted the manuscript, and performed the cell culture experiments. AS performed the tissue-related experiments. TK performed the calcium imaging experiments. WH performed statistical analysis on tissue samples. OH and JK collected human IVD samples and provided clinical expertise. KW-K conceived funding, provided expertise, and helped to draft the manuscript.

## Conflict of Interest Statement

The authors declare that the research was conducted in the absence of any commercial or financial relationships that could be construed as a potential conflict of interest.

## Supplementary Material

Figure S1 The expression of GRP78 in NP and AF of human lumbar intervertebral discs (IVDs). IVDs were collected from donors during spinal surgeries. Gene expression of GRP78 in lumbar NP (*n* = 15 donors) according to the **(A)** degeneration grade and **(B)** type of pathology. Gene expression of GRP78 in lumbar AF (*n* = 12 donors) according to the **(C)** degeneration grade and **(D)** type of pathology. Calculated as 2^−ΔCt^ values (relative to GAPDH). Abbreviations: DDD, degenerative disc disease; DH, disc herniation; AF, annulus fibrosus; NP, nucleus pulposus. The data are not significant.

Figure S2 Cytotoxicity of the used treatments. Cells were isolated from degenerated intervertebral discs and treated for 24 h with 100 and 500 nM thapsigargin (Tg) alone or in combination without **(A)** p38 inhibitor SB2013580 (SB, 10 µM) or **(B)** siRNA against C/EBP homologous protein (CHOP) (siCHOP, 5 nM) and scrambled siRNA negative control (siNC, 5 nM). None of the treatments caused significant cells death, when compared with controls. Data are presented as mean ± SEM relative to control, **p* < 0.05, *n* = 4 (ANOVA with Tukey *post hoc* test).

Figure S3 The effects of transfection reagent Hiperfect (HF) on gene expression of CCAAT-enhancer-binding protein homologous protein (CHOP) and protein release of IL-6. **(E)** The transfection reagent (HF) alone did not induce gene expression of CHOP (*n* = 3 donors). **(F)** HF alone did not induce the release of IL-6 (*n* = 3 donors). Data are presented as mean ± SEM relative to control, **p* < 0.05, *n* = 4 (ANOVA with Tukey *post hoc* test).

Figure S4 The involvement of p38 pathway in IL-1β-induced expression of IL-6, IL-8, and COX-2, and protein release of PGE2. Intervertebral disc (IVD) cells were isolated from degenerated IVDs and treated with 10 ng/mL IL-1β alone or in combination with 10 µM small molecule inhibitor of p38 SB203580 (SB). **(A–C)** Combination of IL-1β and SB-reduced gene expression of IL-6, and COX2, but not IL-8. **(D)** Combination of IL-1β and SB-reduced protein release of PGE2 (*n* = 3). Data are presented as mean ± SEM, **p* < 0.05 (ANOVA with Tukey *post hoc* test).
