# Supplementary figures and images for: p38 MAPK Facilitates Crosstalk Between Endoplasmic Reticulum Stress and IL-6 Release in the Intervertebral Disc
Source: Front Immunol. 2018 Aug 17;9:1706. doi: 10.3389/fimmu.2018.01706 (PMC6107791; doi:10.3389/fimmu.2018.01706)

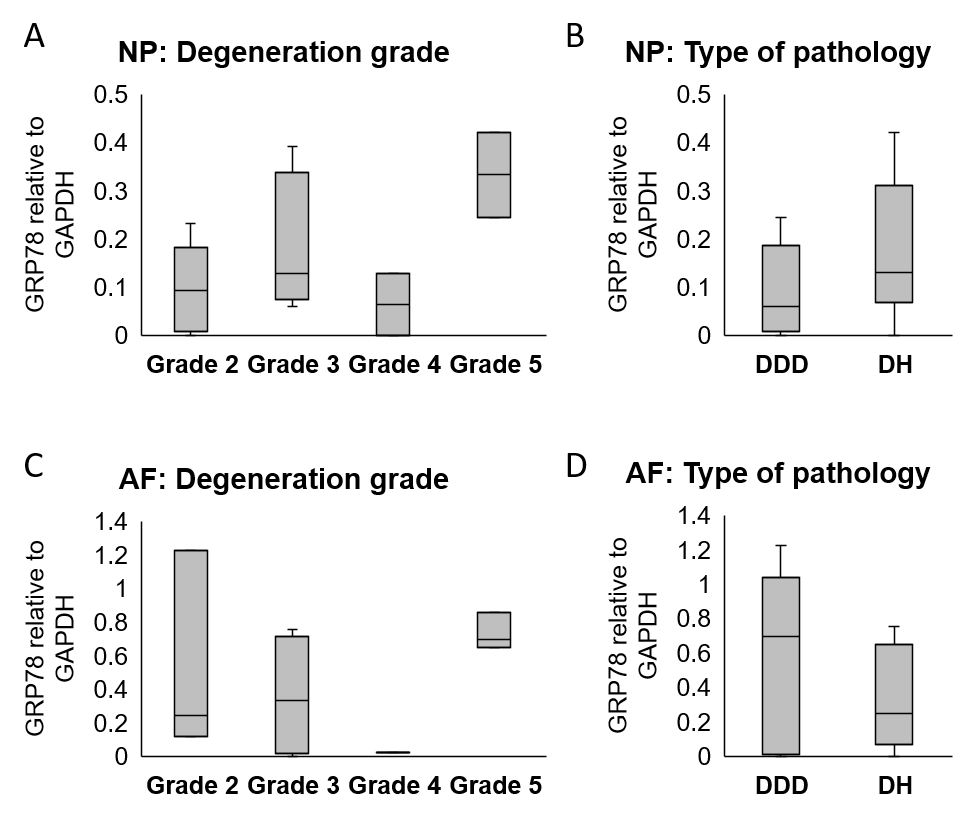

Supplement: Figure S1 — The expression of GRP78 in NP and AF of human lumbar intervertebral discs (IVDs). IVDs were collected from donors during spinal surgeries. Gene expression of GRP78 in lumbar NP (n = 15 donors) according to the (A) degeneration grade and (B) type of pathology. Gene expression of GRP78 in lumbar AF (n = 12 donors) according to the (C) degeneration grade and (D) type of pathology. Calculated as 2−ΔCt values (relative to GAPDH). Abbreviations: DDD, degenerative disc disease; DH, disc herniation; AF, annulus fibrosus; NP, nucleus pulposus. The data are not significant. [file image_1.tif]

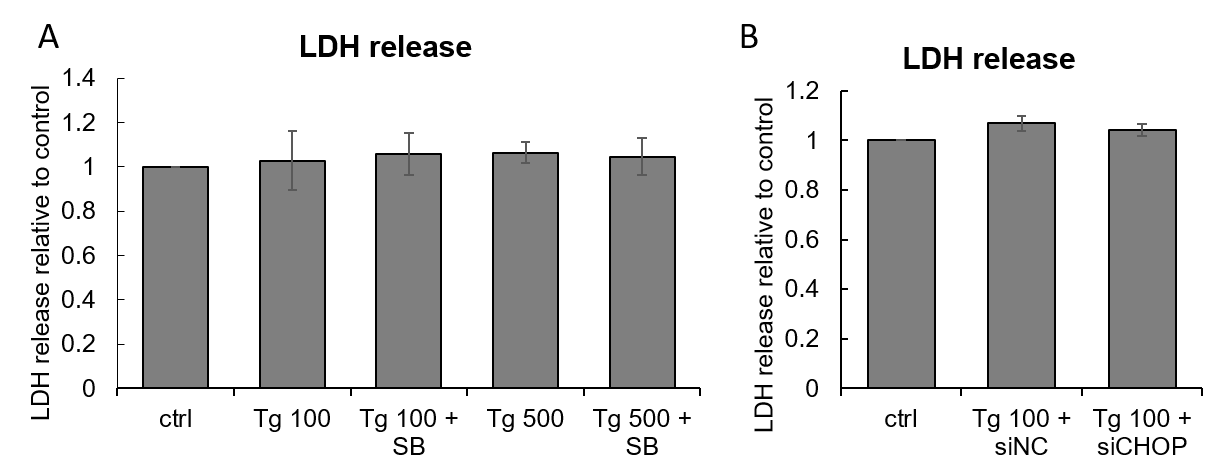

Supplement: Figure S2 — Cytotoxicity of the used treatments. Cells were isolated from degenerated intervertebral discs and treated for 24 h with 100 and 500 nM thapsigargin (Tg) alone or in combination without (A) p38 inhibitor SB2013580 (SB, 10 µM) or (B) siRNA against C/EBP homologous protein (CHOP) (siCHOP, 5 nM) and scrambled siRNA negative control (siNC, 5 nM). None of the treatments caused significant cells death, when compared with controls. Data are presented as mean ± SEM relative to control, *p < 0.05, n = 4 (ANOVA with Tukey post hoc test). [file image_2.tif]

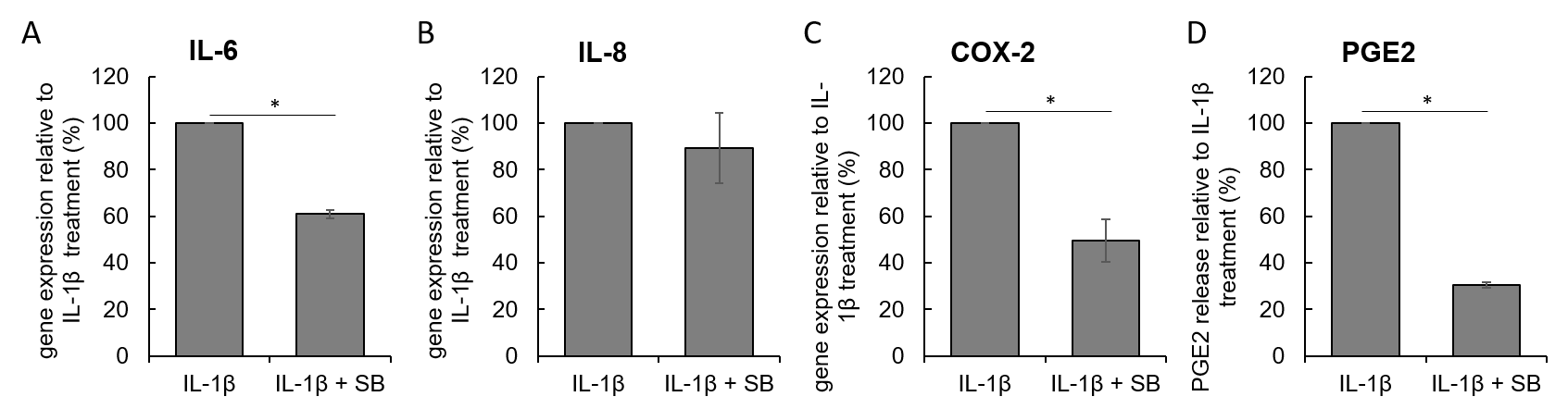

Supplement: Figure S3 — The effects of transfection reagent Hiperfect (HF) on gene expression of CCAAT-enhancer-binding protein homologous protein (CHOP) and protein release of IL-6. (E) The transfection reagent (HF) alone did not induce gene expression of CHOP (n = 3 donors). (F) HF alone did not induce the release of IL-6 (n = 3 donors). Data are presented as mean ± SEM relative to control, *p < 0.05, n = 4 (ANOVA with Tukey post hoc test). [file image_3.tif]

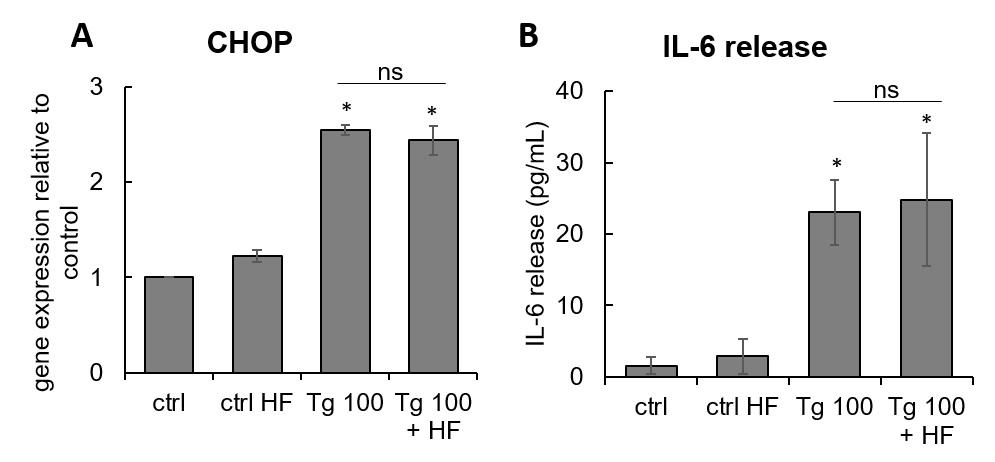

Supplement: Figure S4 — The involvement of p38 pathway in IL-1β-induced expression of IL-6, IL-8, and COX-2, and protein release of PGE2. Intervertebral disc (IVD) cells were isolated from degenerated IVDs and treated with 10 ng/mL IL-1β alone or in combination with 10 µM small molecule inhibitor of p38 SB203580 (SB). (A–C) Combination of IL-1β and SB-reduced gene expression of IL-6, and COX2, but not IL-8. (D) Combination of IL-1β and SB-reduced protein release of PGE2 (n = 3). Data are presented as mean ± SEM, *p < 0.05 (ANOVA with Tukey post hoc test). [file image_4.tif]
