# Supplementary material for: p38 MAPK Facilitates Crosstalk Between Endoplasmic Reticulum Stress and IL-6 Release in the Intervertebral Disc
Source: Front Immunol. 2018 Aug 17;9:1706. doi: 10.3389/fimmu.2018.01706 (PMC6107791; doi:10.3389/fimmu.2018.01706)
Supplement: Supplementary file 5 [file table_1.docx]

***Supplementary table 1****. Significant p-values from all experiments on* ***(A)*** *calcium flux,* ***(B)*** *gene expression and* ***(C)*** *protein release. ns = non-significant; na = not analyzed.*

| **A. Ca flux** | **P values** |
| --- | --- |
| ctrl vs Tg 100 | 0.0000 |
| ctrl vs Tg 500 | 0.0000 |
| Tg 100 vs Tg 500 | 0.0000 |

| **B. Gene expression** | **GRP78** | **CHOP** | **IL-6** | **IL-8** | **COX-2** |
| --- | --- | --- | --- | --- | --- |
| P values gene expression: Tg | | | | | |
| ctrl vs Tg 100 | 0.0016 | 0.0014 | 0.0036 | ns | 0.0000 |
| ctrl vs Tg 500 | 0.0024 | 0.0029 | 0.0012 | 0.0321 | 0.0000 |
| Tg 100 vs Tg 500 | ns | ns | ns | ns | ns |
| P values gene expression: Tg + SB | | | | | |
| Ctrl vs Tg 100 | na | na | 0.0002 | ns | 0.0169 |
| Tg 100 vs Tg + SB | na | na | 0.0007 | ns | ns |
| Ctrl vs Tg 500 | na | na | 0.0002 | ns | 0.0009 |
| Tg 500 vs Tg + SB | na | na | 0.0013 | ns | 0.0510 |
| P values gene expression: cytokines | | | | | |
| Ctrl vs IL-1β 5 | 0.0126 | na | na | na | na |
| Ctrl vs IL-1β 10 | 0.0002 | ns | na | na | na |
| Ctrl vs TNF-α 5 | ns | na | na | na | na |
| Ctrl vs TNF-α 10 | ns | ns | na | na | na |
| P values gene expression: the effects of Hiperfect | | | | | |
| ctrl vs Tg 100 | 0.0000 | na | na | na | na |
| ctrl vs Tg 100 + HF | 0.0000 | na | na | na | na |
| ctrl HF vs Tg 100 | 0.0000 | na | na | na | na |
| ctrl HF vs Tg 100 + HF | 0.0000 | na | na | na | na |

| **C. Protein release** | **IL-6** | **IL-8** | **PGE2** |
| --- | --- | --- | --- |
| P values protein release: Tg | | | |
| Ctrl vs Tg 100 | 0.0010 | ns | ns |
| Ctrl vs Tg 500 | 0.0002 | ns | ns |
| Tg 100 vs Tg 500 | ns | ns | ns |
| P values protein release: Tg + SB | | | |
| Tg 100 vs Tg + SB | 0.0000 | ns | ns |
| Tg 500 vs Tg + SB | 0.0000 | ns | ns |
| P values protein release: Tg + siCHOP | | | |
| Tg 100 vs Tg + siCHOP | 0.0000 | ns | ns |
| P values protein release: the effects of Hiperfect | | | |
| ctrl vs Tg 100 | 0.0147 | na | na |
| ctrl vs Tg 100 + HF | 0.0082 | na | na |
| ctrl HF vs Tg 100 | 0.0454 | na | na |
| ctrl HF vs Tg 100 + HF | 0.0277 | na | na |

| **D. Immunoblotting quantification** | | | | | | | | | | |
| --- | --- | --- | --- | --- | --- | --- | --- | --- | --- | --- |
|  | PERK | IRE1α | GRP78 | CHOP | COX-2 | pJNK | p-p38 | p38 | pNFkB | IκBα |
| Fig 2 | ns | ns | (B) ns  (C) 0.0317 | ns | na | ns | ns | ns | ns | ns |
| Fig 3,4 | na | na | na | na | ns | ns | ns | ns | ns | 0.0277 |
| Fig 5 | ns | ns | 0.0462 | ns | na | na | ns | na | na | na |
| Fig 6 | na | na | ns | na | na | na | na | na | na | na |
